# Alternative trastuzumab dosing strategies in HER2-positive early breast cancer are associated with patient out-of-pocket savings

**DOI:** 10.1038/s41523-022-00393-2

**Published:** 2022-03-14

**Authors:** Po-Hung Hsieh, Alec J. Kacew, Marie Dreyer, Anthony V. Serritella, Randall W. Knoebel, Garth W. Strohbehn, Mark J. Ratain

**Affiliations:** 1 Committee on Clinical Pharmacology and Pharmacogenomics, The University of Chicago, Chicago, IL USA; 2 Pritzker School of Medicine, The University of Chicago, Chicago, IL USA; 3 Department of Medicine, The University of Chicago, Chicago, IL USA; 4 Department of Pharmacy, University of Chicago Medicine, Chicago, IL USA; 5 VA Center for Clinical Management and Research, Ann Arbor, MI USA; 6 VA Ann Arbor Medical Oncology, Ann Arbor, MI USA; 7 Rogel Comprehensive Cancer Center, University of Michigan, Ann Arbor, MI USA; 8 Center for Personalized Therapeutics, The University of Chicago, Chicago, IL USA

**Keywords:** Pharmacoeconomics, Breast cancer, Clinical pharmacology

## Abstract

Patients with breast cancer frequently experience financial hardship, often due to the high costs of anti-cancer drugs. We sought to develop alternative trastuzumab dosing strategies, compare their pharmacokinetic effectiveness to standard dosing, and assess the expected financial implications of transitioning to them. We extracted clinical data from the records of 135 retrospectively identified patients with HER2-positive early breast cancer at a single, urban comprehensive cancer center who were treated with trastuzumab between 2017 and 2019. We performed pharmacokinetic simulations on a range of trastuzumab dose levels and frequencies, assessing efficacy by trough trastuzumab concentration (C_trough_) and population and individual likelihoods of C_trough_ exceeding trastuzumab minimum effective concentration (MEC). We performed deterministic financial modeling to estimate the treatment-associated financial savings from alternative dosing strategies. Trastuzumab maintenance doses of 4 mg/kg every 3 weeks (Q3W) and 6 mg/kg every 4 weeks (Q4W) had nearly identical probabilities of C_trough_ being above MEC as standard of care 6 mg/kg every 3 weeks. In the primary financial analysis, both trastuzumab 4 mg/kg Q3W and 6 mg/kg Q4W were associated with significant drug- and administration-related out-of-pocket cost savings over the duration of therapy, ranging from $765 (neoadjuvant, Q4W) to $2791 (adjuvant, Q4W). In particular, Q4W trastuzumab increased savings related to lost wages and travel cost avoidance. Low-dose and reduced frequency trastuzumab in appropriately selected patients may significantly reduce total drug utilization and meaningfully reduce patient financial toxicity. Prospective clinical trials evaluating low-dose or reduced-frequency administration of therapeutic monoclonal antibodies are warranted and needed.

## Introduction

Invasive breast cancer is the second most common cancer in women, with lifetime incidence of 12% and an estimated 270,000 new cases diagnosed in the United States in 2020^1^. Nearly 55,000 of these newly diagnosed breast cancers express the human epidermal growth factor receptor 2 (HER2), the majority of which are early (i.e., non-metastatic) breast cancers (EBCs)^2^. While the anti-HER2 monoclonal antibody trastuzumab is an evidence-based treatment in the neoadjuvant, adjuvant, or metastatic settings^3,4^, the utilized dose was originally determined in HER2-positive metastatic breast cancer and was never optimized for EBC^5,6^.

Trastuzumab is conventionally administered at a loading dose of 8 mg/kg, followed by maintenance doses of 6 mg/kg every 3 weeks (Q3W) through the end of therapy. Corresponding C_min_ (trough) and C_max_ (peak) concentrations associated with the FDA-labeled Q3W regimen are 47 µg/mL and 179 µg/mL, respectively^5^. However, a trastuzumab concentration of ≥10–20 µg/mL appears sufficient to achieve antibody-dependent cell-mediated cytotoxicity, inhibition of cell proliferation in vitro, and suppression of tumor growth in xenograft murine model^7–11^. Moreover, in the metastatic setting, no differences in progression-free survival (PFS) or overall survival (OS) have been identified between patients with HER2-positive breast cancer who received weight-based trastuzumab Q3W or fixed doses every 4 weeks (Q4W)^12^. Further investigation into the extent to which trastuzumab’s dose can be safely reduced is therefore warranted.

While HER-2-targeted therapy has proven remarkably successful in treating EBC^3^, its associated financial toxicity is significant. Estimated trastuzumab-related costs in the adjuvant setting alone can reach $70,000 per patient, before accounting for lost wages and transportation costs^13^. Patient-experienced financial toxicity subsequently contributed to health-related quality of life losses, medication adherence issues, and inferior outcomes^14–17^. Problems are particularly acute in the developing world, where, despite, trastuzumab being noted as an essential medicine by the World Health Organization, limited access leads to excess cancer-related mortality^18^. Provided efficacy could be maintained, interventional pharmacoeconomic techniques such as lower doses, reduced frequency, or shorter durations may reduce harms related to monoclonal antibodies and improve their value proposition^19,20^.

Pharmacokinetic modeling and simulation are increasingly used to propose new drug dosing regimens and, in some cases, obtain changes to FDA labeling^21,22^. Model-derived alternative dosing regimens can also be used to reduce the clinical and financial toxicities patients experience with their treatment—the intent in this paper. Leveraging previously published population pharmacokinetic models for trastuzumab^23,24^, we performed in silico pharmacokinetic simulations with conventional or alternative dosing regimens of trastuzumab in an institutional population of HER2-positive EBC patients. We hypothesized that the trastuzumab dosing paradigm could be modified so as to enhance trastuzumab’s value proposition to HER2-positive EBC patients, payers, and health systems by limiting the out-of-pocket (OOP) costs patients experience and potentially reducing drug-related adverse events^25^, while maintaining sufficiently high, efficacious therapeutic trough concentrations.

## Results

### Patient population

We identified 196 eligible patients with breast cancer treated with trastuzumab or trastuzumab-anns over the 34-month study period (Table 1). Patients received trastuzumab or biosimilar as single HER-2 inhibiting agent (*n* = 60) or in combination with pertuzumab (*n* = 109). Concurrent chemotherapy was employed in 56% (*n* = 75) of the study population’s regimens, specifically 90% (*n* = 67) in the neoadjuvant setting and 13% (*n* = 8) in the adjuvant setting. Most common concurrent chemotherapy regimens in the neoadjuvant setting included paclitaxel/trastuzumab (54%, *n* = 40), carboplatin/docetaxel/trastuzumab (27%, *n* = 20), and docetaxel/trastuzumab (8%, *n* = 6). The most common concurrent chemotherapy regimen in the adjuvant setting was carboplatin/docetaxel/trastuzumab (8%, *n* = 5) (Table 1).

**Table 1:** Patient clinical and laboratory characteristics (median and interquartile range).

	Neoadjuvant *n* = 74	Adjuvant *n* = 61	EBC*, all patients *n* = 135
Therapy			
*Trastuzumab only*	7	53	60
*Chemotherapy* + *Trastuzumab*	67	8	75
* TH*	40	3	43
* TCH*	20	5	25
* DH*	6	0	6
* CH*	1	0	1
* CTH*	0	0	0
* Eribulin-Trastuzumab*	0	0	0
* Gemcitabine-Trastuzumab*	0	0	0
* Vinorelbine-Trastuzumab*	0	0	0
* Vinorelbine-Trastuzumab-Utomulumab*	0	0	0
Body weight (kg)	73.2 (63.6–87.3)	71.2 (60.9–84.8)	72.2 (61.2–86.3)
AST (IU/L)	19 (15–23)	22 (17–27)	20 (16–25)
Albumin (g/dL)	4.2 (4.0–4.3)	4.1 (3.9–4.2)	4.1 (3.9–4.3)

*EBC refers to ‘early breast cancer’, marked by patients receiving trastuzumab in the neoadjuvant or adjuvant settings. For simplicity, patients who received pertuzumab as part of their chemotherapy + trastuzumab regimen are not separated out from patients who received trastuzumab as the sole HER-2-targeting agent in their regimen.

*TH* paclitaxel + trastuzumab, *TCH* docetaxel + carboplatin + trastuzumab, *DH* docetaxel + trastuzumab, *CH* carboplatin + trastuzumab, *CTH* carboplatin + paclitaxel + trastuzumab.

### Model-predicted pharmacokinetics of trastuzumab

**Fig. 1:**
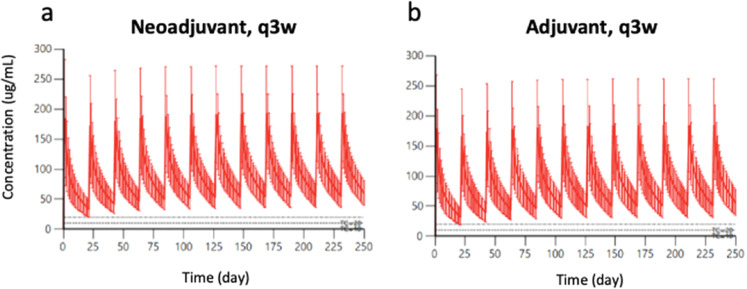
Simulated concentration-time profiles for all HER2-positive early breast cancer patients receiving trastuzumab every 3 weeks. Simulated concentration-time profiles for patients receiving trastuzumab on a q3w schedule in the **a** neoadjuvant or **b** adjuvant. The solid red line indicates median concentration, and the red shaded region represents the 95% CI. Upper dashed line represents concentration = 20 µg/mL. Lower dashed line represents target concentration = 10 µg/mL.

Median trough values predicted by pharmacokinetic simulation are summarized in Fig. 1 and Supplementary Table 2. Median trough of the entire study population, as predicted by pharmacokinetic simulation, was 49 µg/mL (5th–95th percentiles, 23–63 µg/mL). The simulated median trough for the neoadjuvant and adjuvant patients were 55 and 49 µg/mL, respectively. Reference data from manufacturer applications are also included in Supplementary Table 2. Overall, our patient dataset carries similar profiles as the patient population in the manufacturer’s clinical trial.

We evaluated, at the level of the individual patient, the likelihood of trough values being maintained above the minimum effective concentration (Fig. 1). Patients in the neoadjuvant and adjuvant settings had similar trough ranges of 36–64 µg/mL and 32–62 µg/mL, respectively, corresponding to 5th–95th percentiles. (Supplementary Table 2). With conventional dosing, all EBC patients maintained a trough above minimum effective concentration (10 µg/mL). We therefore derived alternative trastuzumab dosing strategies in pharmacokinetic simulations of EBC patients.

### Alternative dosing regimens for EBC patients

#### Development of less frequent dosing regimens

**Fig. 2:**
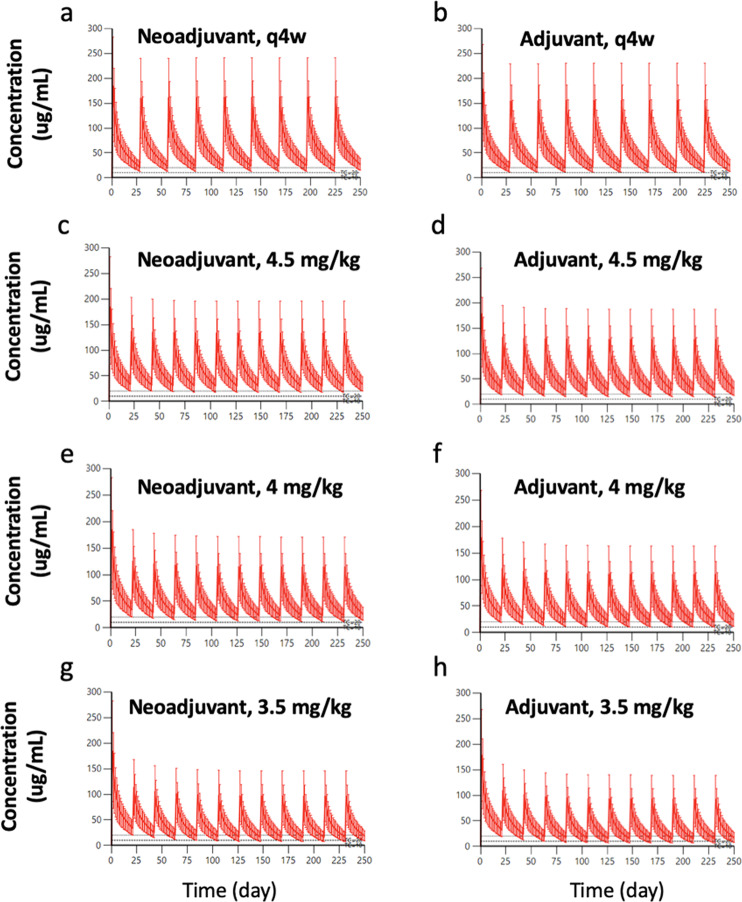
Simulated concentration-time profile for early HER2-positive breast cancer patients receiving interventional pharmacoeconomic dosing of trastuzumab. Less frequently every 4 weeks (Q4W) dosing schedule in the **a** neoadjuvant and **b** adjuvant settings. Every 3 weeks (Q3W) schedule in the **c** neoadjuvant setting with maintenance dose 4.5 mg/kg, **d** adjuvant setting with maintenance dose 4.5 mg/kg, **e** neoadjuvant setting with maintenance dose 4 mg/kg, **f** adjuvant setting with maintenance dose 4 mg/kg, **g** neoadjuvant setting with maintenance dose 3.5 mg/kg, and **h** adjuvant setting with maintenance dose 3.5 mg/kg. In all panels, the solid red line indicates median concentration, and the red shaded region represents 95% CI of the data. Upper dashed line represents concentration = 20 µg/mL. Lower dashed line represents target concentration = 10 µg/mL.

Additional pharmacokinetic simulations were conducted for EBC patients using the 8 mg/kg loading dose followed by 6 mg/kg Q4W. Under this condition, the vast majority of EBC patients maintained a simulated trough ≥10 µg/mL (Fig. 2a, b, Supplementary Table 3). Greater than two-thirds of patients receiving neoadjuvant or adjuvant 6 mg/kg Q4W maintained trough values ≥20 µg/mL (Fig. 2a, b, Supplementary Table 3). We also evaluated regimens in which the Q4W schedule was not implemented until the second or third maintenance dose for EBC patients, as these scenarios would be predicted to have a lower risk of pharmacokinetic failure (Supplementary Table 3).

#### Development of lower-dose regimens

Following an 8 mg/kg loading dose, a range of alternative maintenance doses were evaluated for EBC patients administered using a Q3W schedule: 3, 3.5, 4, 4.5, 5, and 6 mg/kg. Nearly all EBC patients maintained a trough ≥10 µg/mL at doses of 3.5–6 mg/kg (Fig. 2c–h, Supplementary Table 4). Furthermore, the majority of patients maintained a trough ≥20 µg/mL with a maintenance dose of 4 mg/kg. On the other hand, the maintenance dose of 3 mg/kg led to subtherapeutic drug levels in many patients.

### Determinants of trough less than target concentration

EBC patients receiving trastuzumab using extended interval or lower-dose dosing regimens who had any simulated trough concentrations <10 µg/mL were evaluated in further detail. The single greatest driver in these cases was serum albumin concentration <3 g/dL (Supplementary Table 5). Body weight <62 kg (23% of neoadjuvant patients, 28% of adjuvant patient), albumin concentration <4 g/dL (20% of neoadjuvant patients, 36% of adjuvant patients), and AST ≥ 30 IU/L (8% of neoadjuvant patients, 18% of adjuvant patients) were each associated with simulated trough concentration <20 µg/mL (Supplementary Fig. 2, Supplementary Table 6).

### Drug- and administration-related patient-experienced financial toxicity with trastuzumab

#### Primary analysis

Estimated drug- and administration-related cost-sharing payments associated with 6 mg/kg trastuzumab administered Q3W were $3928 (neoadjuvant, *n* = 73) and $9379 (adjuvant, *n* = 60) under non-waste- billing conditions. Drug- and administration-related savings associated with 6 mg/kg Q4W as a percentage of total costs were 14% (neoadjuvant) and 21% (adjuvant). Drug- and administration-related savings associated with 4 mg/kg Q3W were 15% (neoadjuvant) and 20% (adjuvant) (Table 2). Accounting for savings due to travel cost and wage loss avoidance, 6 mg/kg Q4W trastuzumab administration in the adjuvant setting was associated with savings of 21% in the of treatment (Table 2). Drug- and administration-related cost savings were generally greater under Q3W dosing than under Q4W dosing (Supplementary Table 7).

**Table 2:** Per-patient Savings for reduced-frequency trastuzumab compared to current standard-of-care dosing (Q3W, 6 mg/kg).

	Q4W 6 mg/kg	Q3W 4 mg/kg
Neoadjuvant	$765 (14%)	$829 (15%)
Adjuvant	$2791 (21%)	$2611 (20%)

All savings are presented as the discount from the standard of care maintenance trastuzumab 6 mg/kg every 3 weeks for a given clinical scenario and dose (column). Values are savings versus baseline for the comparable clinical setting. Estimates include drug-, administration-, and travel-related cost savings and wage loss avoidance; estimates assume no-waste billing and multi-use vials.

*Q3W* every 3 weeks, *Q4W* every 4 weeks.

#### Sensitivity analyses

Utilization of single-use vials alongside non-waste billing practices was associated with greater patient-experienced expense and absolute savings than multi-use vials and non-waste billing practices (Supplementary Table 8). However, the magnitude of this effect was absent in the 4 mg/kg Q3W condition and was small (<$200 savings over the entire course of therapy) in the 6 mg/kg Q4W condition when compared to multi-use vials. Under conditions of concomitant use of single-use vials and waste billing practices, use of 4 mg/kg Q3W or 6 mg/kg Q4W was associated with increased savings in the time-limited neoadjuvant setting and approximately equal savings in the adjuvant setting, relative to multi-use vials alongside non-waste billing (Supplementary Table 8). Waste billing itself is associated with a greater patient OOP financial liability.

### U.S. health system cost savings

Of the ~50,000 new cases of HER2-positive breast cancer diagnosed in the United States each year, 85% are expected to receive trastuzumab as part of treatment in the neoadjuvant or adjuvant settings for a median of 12 months total. In the base case, assuming conventional 8 mg/kg loading dose, 6 mg/kg every 3 weeks dosing, and patient weight of 75 kg, an estimated 350,625 grams of trastuzumab are provisioned in the U.S. on an annual basis. Resulting annual expected trastuzumab expenditures total $3.24B USD.

Universal application of alternative dosing strategies was associated with marked reductions in total drug unit use and associated savings. Assuming conventional 8 mg/kg loading dose, 4 mg/kg Q3W maintenance dosing, and patient weight of 75 kg, an estimated 242,250 grams of trastuzumab are provisioned in the U.S. on an annual basis, with associated expenditures totaling $2.24B USD ($1.00B savings from base case). Substituting 6 mg/kg Q4W maintenance dosing results in ~274,125 g of trastuzumab used annually, with associated expenditures of $2.53B USD (~$707 M USD savings).

## Discussion

Our analyses demonstrate the potential to safely de-intensify treatment with trastuzumab and its biosimilars (relative to the labeled doses), perhaps reducing total drug units used by as much as 50%. Despite reductions in either maintenance dose frequency from Q3W to Q4W or maintenance dose from 6 to 4 mg/kg, sufficiently high trastuzumab concentrations were almost universally observed. On the basis of these data, appears to be feasible in nearly all patients with HER2-positive EBC, but especially those without medical comorbidities. The financial implications of trastuzumab de-intensification, especially for patients, are especially salient. Estimated OOP savings with reduced frequency trastuzumab were especially notable in the neoadjuvant setting, reaching nearly $1500 per treatment course. In the United States, associated trastuzumab-related savings in EBC alone are estimated to exceed $700 M USD.

Our absolute financial toxicity estimates are in line with prior analyses of real-world data. For individuals diagnosed with breast cancer in 2008–2012, median drug-related OOP costs for trastuzumab-containing regimens was $3381 in the first 18 months of therapy^26^. This is similar to our $3927.76 estimate that includes both drug- and drug administration-related costs in the neoadjuvant setting (Q3W, 6 mg/kg, non-waste billing). Estimates for the OOP costs associated with the adjuvant setting are higher due to longer durations of therapy and continued trastuzumab price increases. Estimates of total EBC trastuzumab-related expenditures in the U.S. (~$3.24B USD) are to a first approximation in line with expectations, given the approximate U.S. share of the global breast cancer therapeutics market, HER2 + EBC epidemiology, and global sales figures for Herceptin® (trastuzumab), totaling ~$6B USD in 2019^27^.

Further dose reductions may be feasible with the incorporation of therapeutic drug monitoring, with dose adjustment based on prior trough concentrations. Given that the manufacturer has stated the minimum target trough concentration is 10–20 µg/mL, we suggest that therapeutic drug monitoring can provide assurance that the trough concentration is acceptable, as well as facilitating even lower doses (or longer treatment intervals) in select patients. EBC patients with normal albumin and hepatic function may be the best candidates for such lower doses. Furthermore, patients with obesity have decreased clearance (normalized to body weight), and thus higher exposure.

De-intensification of trastuzumab in HER2-positive breast cancer remains an area of intense interest^3^. Recently published randomized controlled trials demonstrate the non-inferiority of 6 months of adjuvant trastuzumab compared to 12 months, with the added benefit of lower rates of cardiotoxicity and, we surmise, lower total costs^25^. Similarly, a new paradigm of near-equivalence is emerging, aiming to increase treatment access to the greatest number of patients and reduce financial toxicity, with minimal impact on efficacy^28^. Clinical trial data will likely prove necessary to increase uptake of low-dose trastuzumab. Recently developed multivariable prognostic indices may help identify patients who are at low risk for de-intensification-related adverse outcomes^29^. Using such models may lower a de-intensification clinical trial’s aggregate risk and improve its palatability to patients and physicians.

While oncology awaits a political solution to the ongoing drug pricing crisis, interventional pharmacoeconomic dosing may be a useful strategy in reducing patient-experienced financial toxicity without sacrificing efficacy^20^. In the United States, the approach may help offset drug cost-related risks that are otherwise transferred from insurers to providers in conventional bundled payment arrangements^30^. In both developed and developing economies, interventional pharmacoeconomic dosing may help overcome drug supply limitations while also improving a drug’s value^18^. Interventional pharmacoeconomic dosing can build upon other advances that have improved convenience, drug toxicity, and financial toxicity in recent years (e.g., biosimilar entry, subcutaneous formulation, shorter adjuvant therapy duration)^31–35^. Though the financial analyses demonstrate their potential financial upside (through the avoidance of non-drug-related OOP costs), patients may be hesitant to adopt de-escalated dosing. Prospective clinical trial data will likely be useful in overcoming any gaps in ambiguity, and regardless of their presence it may be justifiable to compensate patients for any theoretical increase in risk under an inclusive shared savings framework^36^.

Not captured in this analysis are the potential benefits of reducing strain on health systems with capacity limitations, whether in the developed or developing world. Delayed initiation of adjuvant trastuzumab is associated with inferior outcomes^37^. To the extent that treatment delays are attributable to over-stressed systems administering drug more frequently than the minimum frequency needed to achieve maximal benefit, less frequent dosing may improve outcomes. In resource-limited settings such as some low- and middle-income countries, low-dose trastuzumab and other interventional pharmacoeconomic techniques could prove particularly useful in enhancing access and reducing financial toxicity, similar to low-dose abiraterone in prostate cancer^38–40^, as well as potentially averting unnecessary deaths^18^.

Notably missing in our analyses are the ~7500 people newly diagnosed with HER2-positive metastatic breast cancer each year in the U.S. Although the number of people with metastatic disease is smaller than the number with EBC, individuals with metastatic disease can remain on HER2-targeting therapy for much longer periods of time. Longer treatment courses in the metastatic setting imply not only higher per-patient cost, but also higher per-patient burden of time and travel. The derived dosing strategies may not apply to many patients in the metastatic setting, owing to increased drug clearance.

This study is not without limitations. First, the pharmacokinetic simulations undergirding the proffered dosing schedules were derived from patients at a single urban academic medical center; thus, the study population may not reflect the makeup of the entire population of HER2-positive EBC patients receiving trastuzumab. Second, the point estimates of expected savings in this paper may have been different had probabilistic modeling techniques been utilized. Third, the point estimates of expected individual-level savings were derived under an assumption of traditional Medicare coverage; patients may have commercial insurance or supplemental co-insurance that limits their OOP liability. Given the emergence of trastuzumab biosimilars, the estimates of health systems savings are likely to be artificially high in absolute terms, though accurate in relative terms. Fourth, we assumed that patients receiving combination treatment could receive both trastuzumab and cytotoxic chemotherapy at the same frequency as trastuzumab (including Q4W instead of Q3W in some cases); the major driver of cost in these cases, however, is the long trastuzumab-only interval. Finally, with the advent of subcutaneous trastuzumab, further study will be needed to assess whether de-intensification of this method of administration is possible.

Altogether, this study demonstrates a roadmap toward de-intensification and waste prevention, not only of trastuzumab in the treatment of HER2-positive EBC but also, more generally, therapeutic monoclonal antibodies in cancer. Adaptive dose or frequency de-escalation trials or randomized, controlled dose-optimization trials with both clinical and pharmacokinetic outcomes exploring the use of maintenance low-dose trastuzumab are warranted, as are further pharmacokinetic studies aimed at de-intensifying monoclonal antibody dosing practices.

## Methods

### Patients

We retrospectively identified all individuals who had received treatment for HER2-positive EBC with trastuzumab or biosimilars at the University of Chicago Medicine (UCM) between January 1, 2017 and October 31, 2019. Patients with incomplete records of trastuzumab administration or any trastuzumab administrations at outside institutions were excluded from the study. We reviewed electronic health records for: Disease and regimen characteristics for each patient in each treatment setting (neoadjuvant or adjuvant), date of first trastuzumab dose, other drugs included in the regimen (e.g., pertuzumab, cytotoxic agents), dose frequency, as well as date and cycle of the last dose (if regimen had been completed) or missed doses. Body weight, aspartate aminotransferase (AST), cancer antigen 15–3 (CA 15–3), albumin as of the day of first trastuzumab dose were collected. In cases in which laboratory values were not available for the day of the first trastuzumab dose, we used the most recent available laboratory value prior to the first trastuzumab dose.

### Pharmacokinetic modeling of trastuzumab

Trastuzumab data were simulated using WinNonlin and NLME with Phoenix Version 8.3 (Certara USA, Inc.). We used a published two-compartment population pharmacokinetic model with parallel linear and nonlinear elimination that included the following covariates: body weight (in kg), AST, and albumin^24^. An R Software package (Version 4.0, http://www.r-project.org/) was used for data assembly, exploratory data analysis, model diagnosis, and simulation.

### Pharmacokinetic simulations of alternative dosing strategies in EBC

We first simulated the standard trastuzumab regimen: 8 mg/kg loading dose followed by 6 mg/kg maintenance dose Q3W in patients with EBC. We then simulated 10 alternative maintenance dosing regimens, a combination of alternative frequencies (Q3W or Q4W) and doses (3, 3.5, 4, 4.5, 5, and 6 mg/kg). We also evaluated regimens in which the alternative dose or schedule was implemented with the second (and all subsequent) or third (and all subsequent) maintenance dose. Pharmacokinetic simulations were performed to 36 weeks to capture 100% steady state. We evaluated schedules based on their achievement of steady-state concentrations of at least 10 µg/mL, in accordance with previously published data, as well as the higher threshold of 20 µg/mL^7,8,41^. Alternative dosing strategies that consistently demonstrated trough concentrations greater than the minimum effective concentration were then pursued in financial modeling studies.

### Statistical analysis

The mean, median, standard deviation, 95% confidence interval, and percentiles of simulated trastuzumab plasma concentrations were calculated using Phoenix Version 8.3 (Certara USA, Inc.) and R Software package (Version 4.0, http://www.r-project.org/).

### Deterministic financial modeling: individual out-of-pocket and nationwide cost estimates

We estimate OOP costs using deterministic financial modeling under varying dosing, patient, treatment setting, and billing conditions. Maintenance trastuzumab was administered in silico to each of the patients identified in the above dataset using one of three dosing cases: 6 mg/kg Q3W (standard of care), 6 mg/kg Q4W, or 4 mg/kg Q3W. The latter two were chosen for their abilities to maintain steady-state concentrations greater than the minimum effective concentration in the previously conducted pharmacokinetic simulation studies. Patients receiving trastuzumab in the neoadjuvant and adjuvant settings received the simulated therapy. We used the actual (i.e., not theoretical or estimated) durations of therapy for the individuals in our cohort to perform our calculations; each individual serves as her own comparator in the analyses.

We derived model inputs from publicly available information: We estimated lost wages^42^, travel distance^43^, mileage rates^44^, parking costs^45^, trastuzumab price, and reimbursement^46,47^, and drug infusion costs (Supplementary Table 1)^48,49^. We considered the lost wages of both the patient and a patient caregiver. Patient travel distance, defined as the distance between the patient’s home address and the main address of the University of Chicago Medicine (UCM), was estimated using the first available search result on GoogleMaps (Alphabet, Inc., Mountain View, CA, USA). Tolls were excluded in mapping preferences. Point estimates of each patient’s expected OOP drug-related financial liability was estimated using a Medicare Part B cost-sharing framework; supplemental co-payment insurances were not considered in this analysis. Two billing methods are used predominantly: non-waste billing and waste billing. Under waste billing provisions, wasted drug is billed to Medicare and is therefore eligible for cost-sharing by the patient. Under non-waste billing, wasted drug is not billed to Medicare and is therefore not eligible for cost-sharing by the patient^50^. Under non-waste billing in which single-use vials are used, the amount of drug is rounded to the nearest measurable dose and/or package size, within 10% (e.g., 768.4 mg is rounded down to 750 mg), and Medicare is billed for the rounded amount of drug administered (750 mg, corresponding to 75 × 10 mg billing units). In the above case, the drug was able to be rounded to the nearest 150 mg vial size (within 10% of target dose), avoiding 132 mg of wasted trastuzumab. If the medication dose cannot be rounded within 10% to the nearest vial size (e.g., a dose of 530 mg), the excess drug (70 mg in the case above) is wasted; Medicare is not billed for the waste^51^. For the purposes of generating cost estimates associated with low-dose trastuzumab under the no-waste billing/single-use vial condition, the 10% tolerance allowed for the FDA-labeled dose was not applied. Multi-use vials can be utilized alongside no-waste billing. In this case, the dose is rounded to the nearest 10 mg dosing unit.

Calculations for cost estimates were as follows:

$$\begin{array}{l}{{Total}}\,{{Out}}{\hbox{-}}{{Of}}{\hbox{-}}{{Pocket}}\,{{Costs}}\\ = \left( {{{{\mathrm{Drug}}}}\,{{{\mathrm{cost}}}}\,{{{\mathrm{sharing}}}}} \right)\\ \quad+ \,\left( {{{{\mathrm{Administration}}}}\,{{{\mathrm{cost}}}}\,{{{\mathrm{sharing}}}}} \right)\\ \quad+ \,\left( {{{{\mathrm{Travel}}}}\,{{{\mathrm{costs}}}}} \right) + \left( {{{{\mathrm{Lost}}}}\,{{{\mathrm{wages}}}}\,{{{\mathrm{per}}}}\,{{{\mathrm{visit}}}}} \right)\end{array}$$

$$\begin{array}{l}{{No}}{\hbox{-}}{{waste}}\,{{billing}},\,{{single}}{\hbox{-}}{{use}}\,{{vial}}\,{{Drug}}\,{{Cost}}\,{{Sharing}}\\ = \left( {{{\mathrm{Number}}}}\,{{{\mathrm{of}}}}\,{{{\mathrm{doses}}}} \right) \\ \quad \times \left( {{{\mathrm{Number}}}}\,{{{\mathrm{of}}}}\,{{{\mathrm{milligrams}}}}\,{{{\mathrm{of}}}}\,{{{\mathrm{trastuzumab}}}}\,{{{\mathrm{per}}}}\,{{{\mathrm{dose}}}}\,{{{\mathrm{per}}}}\,{{{\mathrm{visit}}}},\right.\\ \qquad\left.{{{\mathrm{if}}}}\,{{{\mathrm{possible}}}}\,{{{\mathrm{rounded}}}}\,{{{\mathrm{to}}}}\,{{{\mathrm{nearest}}}}\,{{{\mathrm{whole}}}}\,{{{\mathrm{number}}}}\,{{{\mathrm{of}}}}\,{{{\mathrm{vials}}}}\,{{{\mathrm{within}}}}\,10\% \right) \\ \quad\times \left( {1\,{{{\mathrm{billing}}}}\,{{{\mathrm{unit}}}}/10\,{{{\mathrm{milligrams}}}}\,{{{\mathrm{trastuzumab}}}}} \right)\\ \quad\times \left( {{{{\mathrm{Medicare}}}}\,{{{\mathrm{reimbursement}}}}\,{{{\mathrm{per}}}}\,{{{\mathrm{billing}}}}\,{{{\mathrm{unit}}}}} \right)\\ \quad\times \left( {{{{\mathrm{Medicare}}}}\,{{{\mathrm{patient}}}}\,{{{\mathrm{cost}}}}\,{{{\mathrm{sharing}}}}\,\% } \right)\end{array}$$

$$\begin{array}{l}{{Standard}}\,{{billing}},\,{{single}}{\hbox{-}}{{use}}\,{{vial}}\,{{Drug}}\,{{Cost}}\,{{Sharing}}\\ = \left( {{{{\mathrm{Number}}}}\,{{{\mathrm{of}}}}\,{{{\mathrm{doses}}}}} \right) \times \left( {{{{\mathrm{Rounded}}}}\,{{{\mathrm{number}}}}\,{{{\mathrm{of}}}}\,{{{\mathrm{vials}}}}\,{{{\mathrm{per}}}}\,{{{\mathrm{dose}}}}\,{{{\mathrm{per}}}}\,{{{\mathrm{visit}}}}} \right) \\ \quad \times\left( {{{{\mathrm{Medicare}}}}\,{{{\mathrm{reimbursement}}}}\,{{{\mathrm{per}}}}\,{{{\mathrm{vial}}}}} \right) \times \left( {{{{\mathrm{Medicare}}}}\,{{{\mathrm{patient}}}}\,{{{\mathrm{cost}}}}\,{{{\mathrm{sharing}}}}\,\% } \right)\end{array}$$

$$\begin{array}{l}{{Multi{\hbox{-}}use}}\,{{vial}}\,{{Drug}}\,{{Cost}}\,{{Sharing}} \\= \left( {{{{\mathrm{Number}}}}\;{{{\mathrm{of}}}}\;{{{\mathrm{doses}}}}} \right)\\ \quad\times \left( {{{\mathrm{Number}}}}\;{{{\mathrm{of}}}}\;{{{\mathrm{milligrams}}}}\;{{{\mathrm{of}}}}\;{{{\mathrm{trastuzumab}}}}\;{{{\mathrm{per}}}}\;{{{\mathrm{dose}}}}\right.\\ \qquad \left.{{{\mathrm{per}}}}\;{{{\mathrm{visit}}}},\;{{{\mathrm{rounded}}}}\;{{{\mathrm{to}}}}\;{{{\mathrm{nearest}}}}\;{{{\mathrm{unit}}}} \right) \\ \quad \times \left( {1\;{{{\mathrm{billing}}}}\;{{{\mathrm{unit}}}}/10\;{{{\mathrm{milligrams}}}}\;{{{\mathrm{trastuzumab}}}}} \right)\\ \quad\times \left( {{{{\mathrm{Medicare}}}}\;{{{\mathrm{reimbursement}}}}\;{{{\mathrm{per}}}}\;{{{\mathrm{billing}}}}\;{{{\mathrm{unit}}}}} \right) \\ \quad\times \left( {{{{\mathrm{Medicare}}}}\;{{{\mathrm{patient}}}}\;{{{\mathrm{cost}}}}\;{{{\mathrm{sharing}}}}\;\% } \right)\end{array}$$

$$\begin{array}{l}{{Administration}}\,{{Cost}}\,{{Sharing}}\\ = \left( {{{{\mathrm{Medicare}}}}\;{{{\mathrm{patient}}}}\;{{{\mathrm{cost}}}}\;{{{\mathrm{sharing}}}}\;\% } \right)\\ \quad \times \left( {{{{\mathrm{Time}}}}\;{{{\mathrm{for}}}}\;{{{\mathrm{infusion}}}}\;{{{\mathrm{appointment}}}}} \right)\\ \quad\times \left( {{{{\mathrm{Medicare}}}}\;{{{\mathrm{reimbursement}}}}\;{{{\mathrm{per}}}}\;{{{\mathrm{unit}}}}\;{{{\mathrm{time}}}}} \right)\end{array}$$

$$\begin{array}{l}{{Travel}}\,{{Costs}} = ({{{\mathrm{Number}}}}\;{{{\mathrm{of}}}}\;{{{\mathrm{visits}}}}) \times \left[{{{\mathrm{Parking}}}}\;{{{\mathrm{cost}}}}\;{{{\mathrm{per}}}}\;{{{\mathrm{visit}}}}\right.\\ \qquad\qquad\qquad\quad \left.+ \,({{{\mathrm{Round}}}} {\hbox{-}} {{{\mathrm{trip}}}}\;{{{\mathrm{miles}}}}\;{{{\mathrm{traveled}}}})\right.\\ \qquad\qquad\qquad\quad \left.\times \,({{{\mathrm{IRS}}}}\;{{{\mathrm{reimbursement}}}}\;{{{\mathrm{per}}}}\;{{{\mathrm{mile}}}}\;{{{\mathrm{for}}}}\;{{{\mathrm{medical}}}}\;{{{\mathrm{purposes}}}})\right]\end{array}$$

$${{Lost \ Wages}} = ({\mathrm{Number}} \ {\mathrm{of}} \ {\mathrm{visits}}) \times ({\mathrm{Lost}} \ {\mathrm{wages}} \ {\mathrm{per}} \ {\mathrm{visit}}) \times 2$$

In the primary analysis, we estimated OOP costs assuming that trastuzumab would be administered to a patient who works outside the home at a tertiary referral center (Figure S1). We assumed the utilization of multi-use vials with non-waste-billing as the default billing condition for OOP cost estimation in the primary analysis. As sensitivity analyses, we evaluated local administration of trastuzumab to a patient who is not working outside the home, local administration to a patient who is working outside the home, and tertiary referral center administration to a patient who is not working outside the home. Given nationwide heterogeneity in the use of the Centers for Medicare and Medicaid Services JW signifier, we also examined two alternate billing scenarios: single-use vials with non-waste billing and single-use vials with waste billing.

Nationwide cost-savings estimates were generated from publicly available information regarding incidence, stage at diagnosis, estimated progression-free survival and treatment duration, as well as trastuzumab’s average sales price (ASP)^52,53^. We considered the population of individuals with HER2-positive EBC and estimated median treatment duration by setting (neoadjuvant and adjuvant), under the assumption that treatment is discontinued at the completion of evidence-based time-limited treatment^52,54^. We used Medicare ASP data to estimate the total societal spend associated with this group of patients^53^.

Certain assumptions are necessary. We assumed a treatment duration in the early-stage setting of 12 months. While data from a large, well-conducted randomized, controlled trial demonstrates the non-inferiority of 6 months of adjuvant treatment^25^, cancer treatment guidelines in the U.S. continue to advocate for 12 months of adjuvant anti-HER2 treatment^55^. As commonly administered, this corresponds to 1 cycle of 8 mg/kg and a subsequent 16 cycles of 6 mg/kg every 3 weeks. Total spend under these conditions represents the baseline scenario. Under alternative dosing regiments, the total number of drug units utilized is decreased, though the number of cycles may not be. Using 4 mg/kg every 3 weeks maintenance dosing, the expected numbers of maintenance cycles remain the same as above (16 cycles in neoadjuvant/adjuvant setting). Using 6 mg/kg every 4 weeks maintenance dosing, the expected numbers of maintenance cycles are less (13 cycles in neoadjuvant/adjuvant setting). We used Medicare ASP data to estimate the total societal spend associated with this group of patients^53^. Contracted prices for our institution are not made publicly available; ASP captures drug price net of the majority of rebates and discounts.

Calculations for cost estimates were as follows:

$$\begin{array}{*{20}{l}}{Total \,Spend}={\left[\left[({\rm{Number}}\,{\rm{of}}\,{\rm{Newly}}\,{\rm{Diagnosed}}\,{\rm{Breast}}\,{\rm{Cancer}}\,{\rm{Cases}})\right.\right.}\\{ \times \,(\% \,{\rm{of}}\,{\rm{Cases}}\,{\rm{HER}}2 {\hbox{-}} {\rm{Positive}})\, \times \, (\% \,{\rm{of}}\,{\rm{HER}}2 {\hbox{-}} {\rm{Positive}}\,{\rm{Cases}}\,{\rm{Early}} {\hbox{-}} {\rm{Stage}})}\\{\left. \times \,(8\,{\rm{mg}}/{\rm{kg }} \ast {\rm{ weight}}\, + \,{\rm{Maintenance}}\,{\rm{dose}}\, \ast \,{\rm{weight}}\, \ast \,{\rm{number}}\,{\rm{of}}\,{\rm{treatments}})\right]/(10\,{\rm{mg}}\,{\rm{per}}\,{\rm{dosing}}\,{\rm{unit}})}\\{\left. \times \,({\rm{Average}}\,{\rm{Sales}}\,{\rm{Price}}\,{\rm{per}}\,10\,{\rm{mg}}\,{\rm{Dosing}}\,{\rm{Unit}})\right]}\end{array}$$

$$\begin{array}{l}{{Savings}} = {{{\mathrm{Baseline}}}}\;{{{\mathrm{scenario}}}}\;{{{\mathrm{expenditures}}}}\\ \qquad\qquad\quad -{{{\mathrm{Alternative}}}}\;{{{\mathrm{dosing}}}}\;{{{\mathrm{scenario}}}}\;{{{\mathrm{expenditures}}}}\end{array}$$

### Reporting summary

Further information on research design is available in the Nature Research Reporting Summary linked to this article.

## Supplementary information

Reporting Summary

Resupplied SI file

## Data Availability

The datasets generated and analyzed during the current study are not publicly available due to their containing individually identifiable information. A redacted dataset is available from the corresponding author on reasonable request.

## Code availability

Relevant code and spreadsheet formulae for population pharmacokinetic and financial analyses are available from the authors.
