# Supplementary material for: Alternative trastuzumab dosing strategies in HER2-positive early breast cancer are associated with patient out-of-pocket savings
Source: NPJ Breast Cancer. 2022 Mar 14;8:32. doi: 10.1038/s41523-022-00393-2 (PMC8921207; doi:10.1038/s41523-022-00393-2)
Supplement: Supplementary file 2 — Resupplied SI file [file 41523_2022_393_MOESM2_ESM.pdf]

SUPPLEMENTARY FIGURE AND TABLE LEGENDS

Supplementary Figure 1. Clinical scenarios utilized for financial modeling. Four common clinical scenarios were included: (A) Patient not currently employed outside the home who receives therapy locally and without any transportation or parking cost; (B) Patient currently employed outside the home who receives therapy locally and without any transportation or parking costs; (C) Patient not currently employed outside the home who receives therapy at a referral center and thus experiences transportation and parking costs; and (D) Patient currently employed outside the home who receives therapy at a referral center and thus experiences transportation and parking costs. Abbreviation: RT, round-trip.

Supplementary Table 1. Model input and sources. Inputs were derived from publicly available information. IRS, Internal Revenue Service of the United States of America; USD, US dollars. References refer to those in the manuscript body.

Supplementary Table 2. Median trough concentrations predicted by pharmacokinetic simulations. Table values represent median trastuzumab trough concentration (5th -95th percentiles) in patients with HER2-positive breast cancer, expressed in µg/mL. ‡ Full prescribing information in FDA approval package (application number: 103792Orig1s5337, Genentech, 2017). * Patients with early breast cancer (EBC) corresponds to patients receiving trastuzumab in the neoadjuvant or adjuvant settings.

Supplementary Table 3. Estimated trough trastuzumab concentrations using Q4W maintenance dosing in patients with early breast cancer. Values in the table represent median with 95% CI of simulated trough concentration, expressed in µg/mL.

Supplementary Table 4. Simulated trastuzumab trough with different q3w maintenance doses. Values represent C_{trough} in µg/mL (median and 95% confidence interval).

Supplementary Table 5. Associated laboratory findings in patients with trastuzumab trough concentrations < 10 μg/mL. Baseline laboratory values in patients with simulated trough concentrations less than minimum effective concentration (10 μg/mL). AST, aspartate aminotransferase; ALB, albumin.

Supplementary Table 6. Associated laboratory findings in patients with trastuzumab trough concentrations < 20 μg/mL. Baseline laboratory values in patients with simulated trough concentrations less than 20 μg/mL. AST, aspartate aminotransferase; ALB, albumin.

Supplementary Figure 2. Simulated concentration-time profiles for individual patients with early breast cancer. Simulated concentration-time profile for each patient with (a) every 4 weeks (Q4W) schedule and 6 mg/kg maintenance dose in the neoadjuvant setting, (b) Q4W schedule and 6 mg/kg maintenance dose in the adjuvant setting, (c) every 3 weeks (Q3W) schedule and 4 mg/kg maintenance dose in the neoadjuvant setting, and (d) Q3W schedule and 4 mg/kg maintenance dose in the adjuvant setting. The solid red lines indicate simulated concentration for each patient. Upper dashed line represents concentration = 20 μg/mL. Lower dashed line represents target concentration = 10 μg/mL.

Supplementary Table 7. Estimated savings associated with trastuzumab therapy under alternative dosing schedules. All savings are presented as the discount from the standard of care maintenance trastuzumab 6 mg/kg every 3 weeks for a given clinical scenario and dose (column). In the top panel, savings are presented in USD saved compared to standard of care. Bottom panel represents savings as a percentage discount off the costs associated with standard of care. Values are savings versus baseline for the comparable clinical setting. Abbreviations: Q3W, every 3 weeks; Q4W, every 4 weeks; IVPE, interventional pharmacoeconomics.

Supplementary Table 8. Estimated drug, administration, and other out-of-pocket costs experienced by patients with HER2-positive breast cancer receiving trastuzumab therapy under alternative dosing schedules. Trastuzumab-related costs assumed by patients with HER2-positive breast cancer receiving trastuzumab therapy: (A) 6 mg/kg every 3 weeks (Q3W) (standard of care) (non-waste billing, multi-use

vials), (B) 6 mg/kg every 4 weeks (Q4W) (non-waste billing, multi-use vials), (C) 4 mg/kg Q3W (non-waste billing, multi-use vials), (D) 6 mg/kg Q3W (standard of care) (non-waste billing, single-use vials), (E) 6 mg/kg Q4W (non-waste billing, single-use vials), (F) 4 mg/kg Q3W (non-waste billing, single-use vials), (G) 6 mg/kg Q3W (standard of care) (waste billing, single-use vials), (H) 6 mg/kg Q4W (waste billing, single-use vials), and (I) 4 mg/kg Q3W (waste billing, single-use vials). Formulae used to calculate savings are summarized in **Methods**. Abbreviations: OOP, out-of-pocket; Q3W, every three weeks; Q4W, every four weeks.

Supplementary Figure 1. Clinical scenarios utilized for financial modeling.

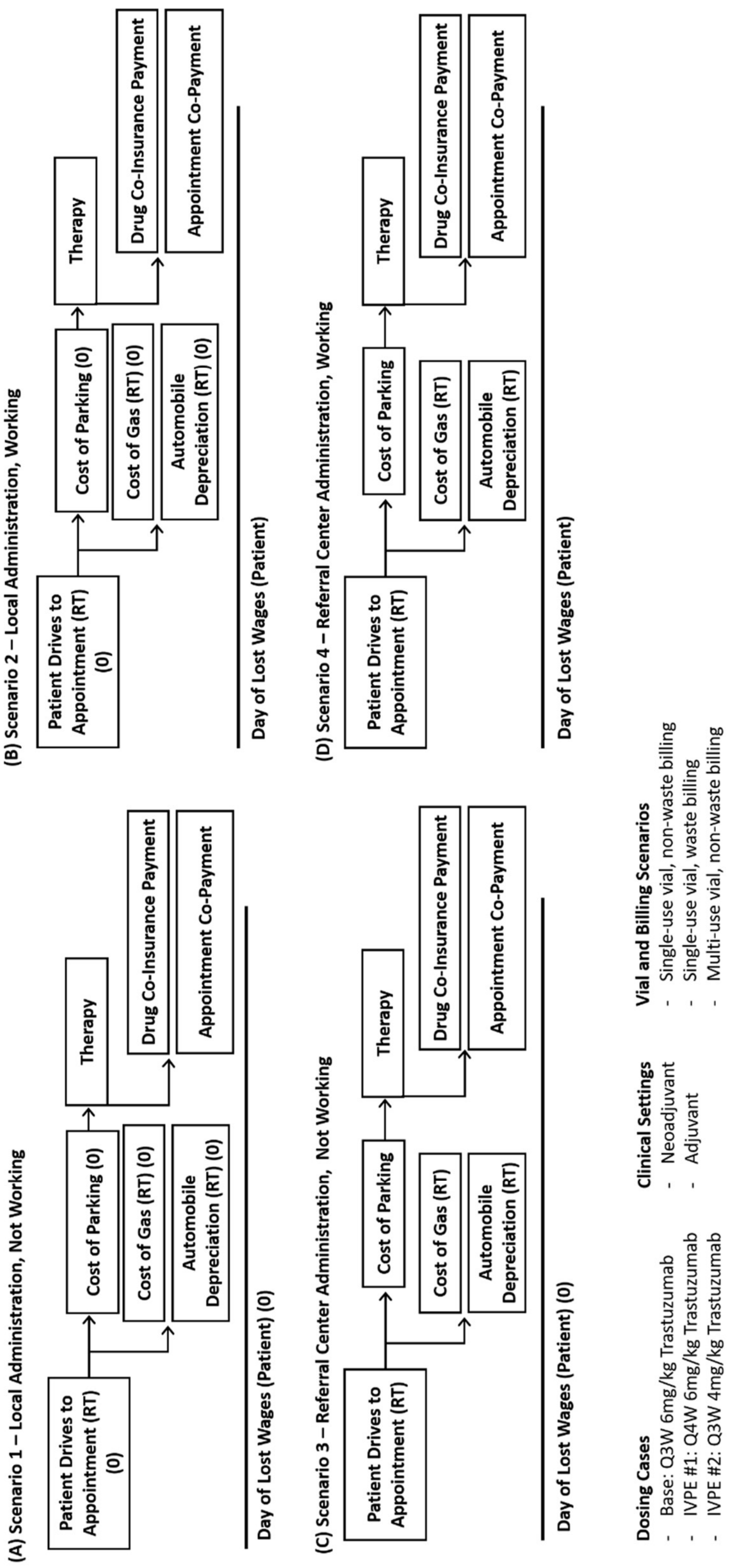

Supplemental Table 1. Model input and sources.

Input	Value	Reference
Cost of Parking ($ per Appointment)	$15.00	45
Median Daily Wage in United States ($ per Day)	$187.20	42
Cost per Mile for Medical Purposes ($ per Mile)	$0.17	44
Trastuzumab Single-Use Vial Size (mg)	150	47
Trastuzumab Medicare Drug Reimbursement per 10mg	$99.68	47
Medicare Reimbursement per Vial	$1,495.13	*Calculated*
Time for First Trastuzumab Infusion (min)	90	48,49
Time for Subsequent Trastuzumab Infusion(s) (min)	30	48,49
Medicare Infusion Reimbursement (up to 1 hr)	$142.55	47
Medicare Infusion Reimbursement (per hr for subsequent hrs)	$30.68	47
Medicare Cost-Sharing	20%	46

Supplemental Table 2. Median trough concentrations predicted by pharmacokinetic simulations.

Setting	UCM, neoadjuvant n = 74	UCM, adjuvant n = 61	UCM, metastatic n = 61	UCM, EBC* n = 135	UCM, all patients n = 196	Manufacturer n = 1195
Trough (µg/mL)	55 (36-64)	49 (32-62)	41 (15-62)	52 (35-64)	49 (23-63)	47 (5-115)‡
% Trough ≥ 10 µg/mL	100 %	100 %	98 %	100 %	99 %	N/A
% Trough ≥ 20 µg/mL	100 %	98 %	92 %	99 %	97 %	N/A

Supplemental Table 3. Estimated trough trastuzumab concentrations using Q4W maintenance dosing in patients with early breast cancer.

q4w dosing timing	Neoadjuvant (95% CI)	% trough ≥ 10 μg/mL (≥ 20 μg/mL)	Adjuvant (95% CI)	% trough ≥ 10 μg/mL (≥ 20 μg/mL)
At the first dose	25 (13 - 35)	99% (77%)	22 (11 - 33)	97% (71%)
At the second dose	26 (14 - 36)	99% (80%)	23 (11 - 34)	97% (75%)
At the third dose	27 (14 - 37)	99% (84%)	23 (12 - 34)	97% (75%)

Supplemental Table 4. Simulated trastuzumab trough with different Q3W maintenance doses.

Maintenance doses	Neoadjuvant, (95% CI)	% trough ≥ 10 µg/mL (≥ 20 µg/mL)	Adjuvant, (95% CI)	% trough ≥ 10 µg/mL (≥ 20 µg/mL)
Standard 6 mg/kg	55 (34-70)	100% (100%)	49 (30-67)	100% (98%)
5 mg/kg	40 (23-52)	100% (99%)	35 (20-50)	100% (97%)
4.5 mg/ kg	32 (18-44)	100% (92%)	28 (15-41)	98% (92%)
4 mg/kg	25 (13-35)	100% (77%)	22 (11-33)	97% (70%)
3.5 mg/kg	18 (9-27)	93% (45%)	16 (7-25)	92% (30%)
3 mg/kg	13 (5-20)	76% (1%)	11 (4-18)	69% (2%)

Supplemental Table 5. Associated laboratory findings in patients with trastuzumab trough concentrations < 10 μg/mL.

Patient ID	C_{trough} (μg/mL)	AST (IU/L)	ALB (g/dL)
Loading 8 mg/kg, Q4W maintenance 6 mg/kg, neoadjuvant			
25	9	10	2.1
Loading 8 mg/kg, Q4W maintenance 6 mg/kg, adjuvant			
5	8	45	2.9
21	6	32	2.9
Loading 8 mg/kg, Q3W maintenance 4 mg/kg, neoadjuvant			
Loading 8 mg/kg, Q3W maintenance 4 mg/kg, adjuvant			
5	8	45	2.9
21	6	32	2.9

Supplemental Table 6. Associated laboratory findings in patients with trastuzumab trough concentrations < 20 μg/mL.

Patient ID	C_{trough} (μg/mL)	AST (IU/L)	ALB (g/dL)
Loading 8 mg/kg, Q4W maintenance 6 mg/kg, neoadjuvant			
11	17	30	3.8
12	14	29	4.4
13	15	28	3.9
18	17	13	3.3
21	14	14	4.4
25	9	10	2.1
27	14	24	4.5
31	15	28	3.3
34	17	34	3.7
37	18	28	3.9
42	14	53	3.7
49	19	49	4.6
52	19	23	4.1
67	19	23	4.2
68	19	20	4.2
70	14	19	4.1
72	18	18	4.2
Loading 8 mg/kg, Q4W maintenance 6 mg/kg, adjuvant			
5	8	45	2.9
8	16	61	4.0
10	17	30	3.8
11	12	26	3.9
17	19	24	3.7
18	13	18	4.3
20	13	12	3.1
21	6	32	2.9
22	19	23	3.5
26	18	20	4.0
27	17	34	3.8
34	19	18	3.9
38	18	25	3.9
41	19	23	4.1
44	18	35	4.5
50	19	23	3.7
54	18	30	4.0
57	18	23	4.0
Loading 8 mg/kg, Q3W maintenance 4 mg/kg, neoadjuvant			
11	18	30	3.8
12	13	29	4.4
13	15	28	3.9
18	18	13	3.3
21	13	14	4.4
25	11	10	2.1
27	13	24	4.5
31	16	28	3.3
34	17	34	3.7
37	18	28	3.9
42	15	53	3.7
49	19	49	4.6
52	19	23	4.1

Patient ID	C_{trough} (μg/mL)	AST (IU/L)	ALB (g/dL)
67	19	23	4.2
68	18	20	4.2
70	13	19	4.1
72	18	18	4.2
Loading 8 mg/kg, Q3W maintenance 4 mg/kg, adjuvant			
5	8	45	2.9
8	17	61	4.0
10	17	30	3.8
11	12	26	3.9
18	12	18	4.3
20	13	12	3.1
21	6	32	2.9
26	17	20	4.0
27	17	34	3.8
34	19	18	3.9
38	18	25	3.9
40	19	33	4.5
41	19	23	4.1
44	18	35	4.5
50	19	23	3.7
54	18	30	4.0
57	17	23	4.0
59	19	15	4.2

Supplementary Figure 2. Simulated concentration-time profiles for individual patients with early breast cancer.

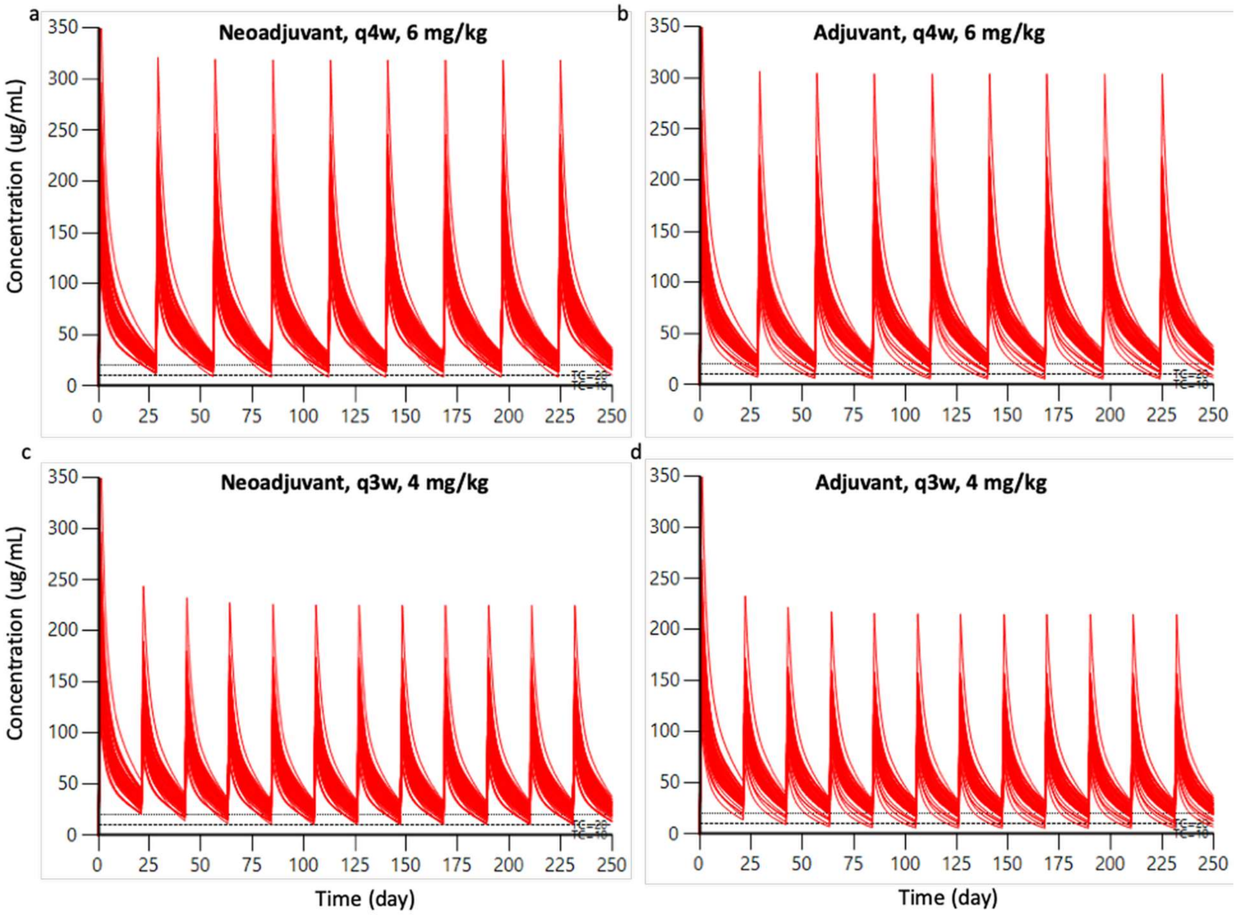

Supplemental Table 7. Savings for IVPE trastuzumab compared to current standard-of-care dosing.

	Q4W 6mg/kg				Q3W 4mg/kg			
	Drug- and Administration-Related Cost Savings	Drug- and Administration-Related Cost Savings and Wage Loss Avoidance	Drug-, Administration-, and Travel-Related Cost Savings	Drug-, Administration-, and Travel-Related Cost Savings and Wage Loss Avoidance	Drug- and Administration-Related Cost Savings	Drug- and Administration-Related Cost Savings and Wage Loss Avoidance	Drug-, Administration-, and Travel-Related Cost Savings	Drug-, Administration-, and Travel-Related Cost Savings and Wage Loss Avoidance
**Absolute per-patient savings of IVPE vs. baseline (Q3W 6mg/kg) ($ USD)**								
**Neoadjuvant, no-waste billing, multi-use vial**	$367	$742	$391	$765	$829	$829	$829	$829
**Adjuvant, no-waste billing, multi-use vial**	$1,605	$2,728	$1,667	$2,791	$2,611	$2,611	$2,611	$2,611
**Neoadjuvant, no-waste billing, single-use vial**	$356	$730	$380	$754	$826	$826	$826	$826
**Adjuvant, no-waste billing, single-use vial**	$1,703	$2,826	$1,766	$2,889	$2,611	$2,611	$2,611	$2,611
**Neoadjuvant, standard billing, single-use vial**	$897	$1,271	$921	$1,295	$1,196	$1,196	$1,196	$1,196
**Adjuvant, standard billing, single-use vial**	$1,951	$3,074	$2,014	$3,137	$2,884	$2,884	$2,884	$2,884
**Relative per-patient savings of IVPE vs. baseline (Q3W 6mg/kg) (%)**								
**Neoadjuvant, no-waste billing, multi-use vial**	9%	14%	10%	14%	21%	15%	21%	15%
**Adjuvant, no-waste billing, multi-use vial**	17%	21%	17%	21%	28%	20%	27%	20%
**Neoadjuvant, no-waste billing, single-use vial**	9%	13%	9%	14%	21%	15%	21%	15%
**Adjuvant, no-waste billing, single-use vial**	18%	22%	18%	22%	28%	20%	27%	20%
**Neoadjuvant, standard billing, single-use vial**	18%	20%	19%	20%	25%	19%	24%	19%
**Adjuvant, standard billing, single-use vial**	18%	21%	18%	21%	26%	20%	26%	19%

Key
= greatest savings
= least savings

Supplemental Table 8. Patient-experienced drug, administration, and other out-of-pocket costs with trastuzumab therapy with different dosing schedules.

(A) Median estimated costs for standard-of-care Q3W 6mg/kg dosing (non-waste billing, multi-use vials)

	Drug and administration cost sharing	Lost wages	Travel costs	Total OOP costs
Neoadjuvant (n=73)	$3,927.76	$748.80	$91.84	$5,552.78
Adjuvant (n=60)	$9,378.81	$1,872.00	$210.99	$13,768.73

(B) Median estimated costs for Q4W 6mg/kg dosing (non-waste billing, multi-use vials)

	Drug and administration cost sharing	Lost wages	Travel costs	Total OOP costs
Neoadjuvant (n=73)	$3,560.36	$561.60	$63.54	$4,770.29
Adjuvant (n=60)	$7,773.93	$1,310.40	$155.20	$10,765.23

(C) Median estimated costs for Q3W 4mg/kg dosing (non-waste billing, multi-use vials)

	Drug and administration cost sharing	Lost wages	Travel costs	Total OOP costs
Neoadjuvant (n=73)	$3,099.07	$748.80	$91.84	$4,715.51
Adjuvant (n=60)	$6,767.33	$1,872.00	$210.99	$11,127.34

(D) Median estimated costs for standard-of-care Q3W 6mg/kg dosing (non-waste billing, single-use vials)

	Drug and administration cost sharing	Lost wages	Travel costs	Total OOP costs
Neoadjuvant (n=73)	$3,916.40	$748.80	$91.84	$5,492.57
Adjuvant (n=60)	$9,378.81	$1,872.00	$210.99	$13,505.16

(E) Median estimated costs for Q4W 6mg/kg dosing (non-waste billing, single-use vials)

	Drug and administration cost sharing	Lost wages	Travel costs	Total OOP costs
Neoadjuvant (n=73)	$3,560.36	$561.60	$63.54	$4,770.29
Adjuvant (n=60)	$7,675.65	$1,310.40	$155.20	$10,645.02

(F) Median estimated costs for Q3W 4mg/kg dosing (non-waste billing, single-use vials)

	Drug and administration cost sharing	Lost wages	Travel costs	Total OOP costs
Neoadjuvant (n=73)	$3,090.49	$748.80	$91.84	$4,715.51
Adjuvant (n=60)	$6,767.33	$1,872.00	$210.99	$11,107.41

(G) Median estimated costs for standard-of-care Q3W 6mg/kg dosing (waste billing, single-use vials)

	Drug and administration cost sharing	Lost wages	Travel costs	Total OOP costs
Neoadjuvant (n=73)	$4,876.07	$748.80	$91.84	$6,071.09
Adjuvant (n=60)	$10,949.39	$1,872.00	$210.99	$15,035.09

(H) Median estimated costs for Q4W 6mg/kg dosing (waste billing, single-use vials)

	Drug and administration cost sharing	Lost wages	Travel costs	Total OOP costs
Neoadjuvant (n=73)	$3,978.99	$561.60	$63.54	$5,178.30
Adjuvant (n=60)	$8,998.43	$1,310.40	$155.20	$11,829.83

(I) Median estimated costs for Q3W 4mg/kg dosing (waste billing, single-use vials)

	Drug and administration cost sharing	Lost wages	Travel costs	Total OOP costs
Neoadjuvant (n=73)	$3,679.97	$748.80	$91.84	$5,407.23
Adjuvant (n=60)	$8,065.89	$1,872.00	$210.99	$12,343.86
